# miRNAs in stem cell-derived extracellular vesicles for acute kidney injury treatment: comprehensive review of preclinical studies

**DOI:** 10.1186/s13287-019-1371-1

**Published:** 2019-09-03

**Authors:** Si-Yang Wang, Quan Hong, Chao-Yang Zhang, Yuan-Jun Yang, Guang-Yan Cai, Xiang-Mei Chen

**Affiliations:** Department of Nephrology, Chinese PLA General Hospital, Chinese PLA Institute of Nephrology, State Key Laboratory of Kidney Diseases, National Clinical Research Center for Kidney Diseases, Beijing Key Laboratory of Kidney Diseases, Beijing, 100853 China

**Keywords:** Stem cell, microRNA, Acute kidney injury

## Abstract

Stem cell therapy has been applied in many fields. Basic and clinical studies on stem cell therapy for acute kidney injury (AKI) have been conducted. Stem cells have been found to exert renal protection through a variety of mechanisms, such as regulating the immune system and secreting growth factors, cytokines, and extracellular vesicles (EVs). Among them, EVs are considered to be important mediators for stem cell protection because they contain various biological components, including microRNAs (miRNAs). miRNAs are a class of small RNAs that function in posttranscriptional gene regulation. A number of studies have confirmed that miRNAs in stem cell-derived EVs can protect from AKI. miRNAs can enter the injured renal tissue through EVs released from stem cells, thereby exerting anti-inflammatory, anti-apoptotic, anti-fibrotic, and pro-angiogenesis effects on AKI. However, the stem cell sources and AKI models used in these studies have differed. This article will summarize the miRNAs that play a role in kidney protection in stem cell EVs and clarifies the treatment characteristics and mechanisms of different miRNAs. This may provide a reference for clinical practice for acute and chronic kidney diseases.

## Background

Extracellular vesicles (EVs) are membrane-enclosed microvesicles released by cells. EVs carry various biological molecules, including microRNAs (miRNAs), proteins, mRNAs, long noncoding RNAs, DNA, and lipids, and deliver them to recipient cells [1]. EVs were first thought to be a mechanism for cells to remove metabolic waste and maintain cellular homeostasis. Later, they were found to play a key role in cell communication. It is currently believed that EVs are an important route for cellular communication and can be released and endocytosed by most types of cells [2].

The miRNAs in EVs are important regulators of intercellular communication. miRNAs, which are widely present in eukaryotes, are a class of noncoding, single-stranded, small-molecule RNAs approximately 18–25 nucleotides in length. They can inhibit target mRNA translation or induce target mRNA degradation through partial or full complementarity to the 3′ untranslated region of target mRNAs, thereby enabling posttranscriptional gene regulation [3]. Stem cells have been shown to play a therapeutic role in acute kidney injury (AKI) by secreting EVs rich in miRNAs, and the mechanism has been extensively studied. The protective effect of EVs on kidney injury can be reversed by miRNA deficiency caused by knocking out the ribonuclease III Drosha gene in mesenchymal stem cells (MSCs) [4]. However, stem cells from different sources may have different miRNAs that function in different types of AKI. This study will summarize the miRNAs in stem cell-derived EVs with confirmed therapeutic effects on AKI.

### Stem cell-derived EVs and miRNAs

Almost all cell types in the human body can secrete EVs that subsequently enter circulation. EVs transmit signals to distant cells via paracrine mechanisms to mediate intercellular communication and participate in a variety of physiological and pathological processes. Therefore, EVs are considered important information carriers that regulate the gene expression and phenotypes of adjacent or distal recipient cells through paracrine mechanisms [5].

EVs can mediate the communication between stem cells and damaged cells. On the one hand, EVs secreted by stem cells can act on damaged cells. For example, EVs secreted by mouse embryonic stem cells can reprogram hematopoietic progenitor cells by transmitting transcription factors, proteins, and mRNAs [6]. On the other hand, studies have shown that damaged cells can secrete EVs to regulate stem cell functions [7]. EVs derived from injured cells can transmit specific signals to stem cells and trigger stem cell differentiation, which may be another potential mechanism for EVs to repair pathological tissues [8].

**Fig. 1:**
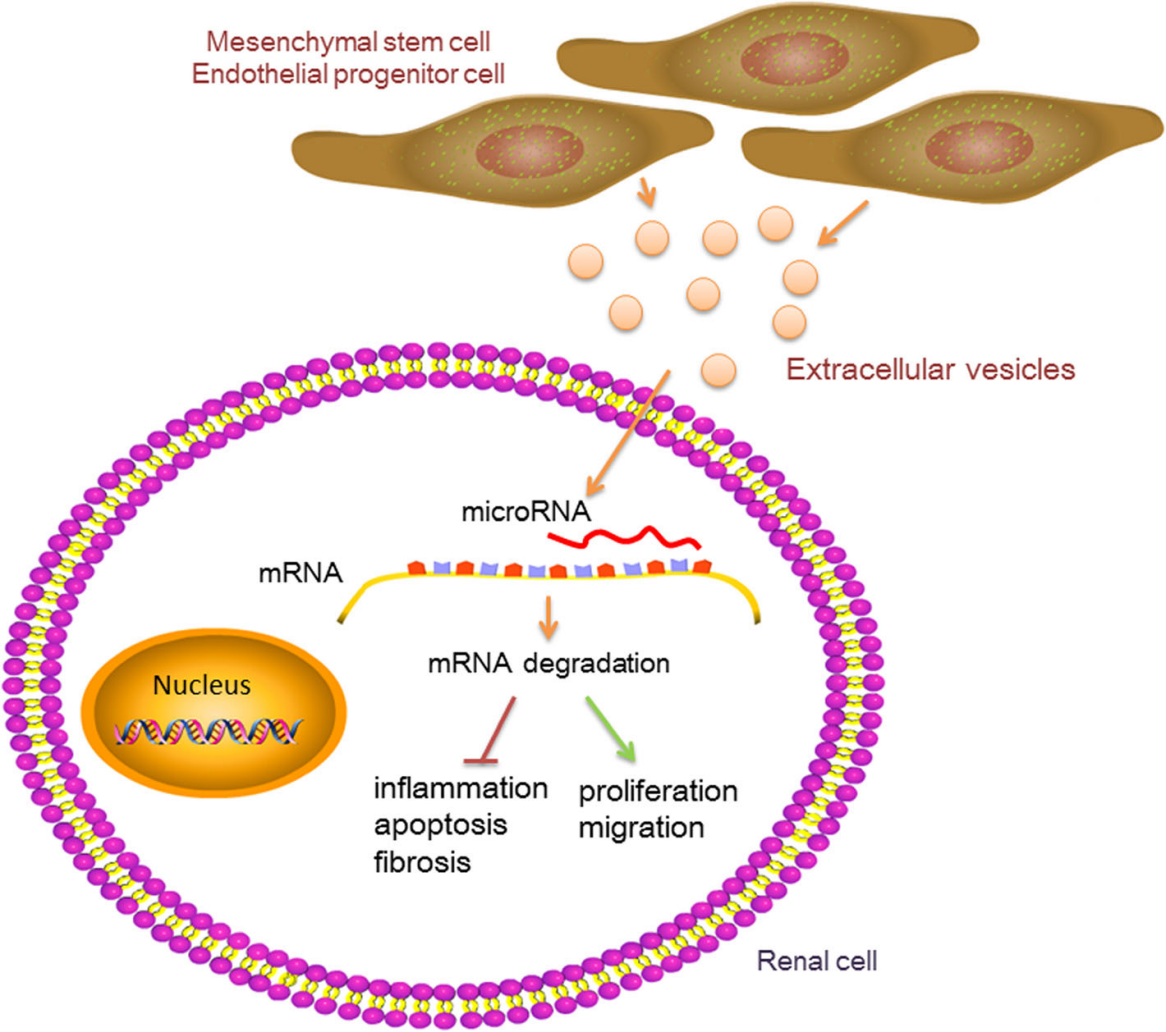
The mechanism of miRNAs in stem cell-derived extracellular vesicles for acute kidney injury. Extracellular vesicles secreted by stem cells carry miRNAs and deliver them into renal cells. These miRNAs inhibit target mRNA translation or induce target mRNA degradation through complementarity to the region of target mRNAs. This process can play an anti-inflammatory, anti-apoptotic, and anti-fibrotic effects on AKI

Paracrine mechanisms are one of the important modes through which stem cells play a therapeutic role in AKI. The therapeutic effect of EVs on AKI has been extensively studied (Fig. 1). Many studies have demonstrated the therapeutic effect of EV injection alone on AKI. Various substances contained in EVs—such as angiogenin, hepatocyte growth factor (HGF), vascular endothelial growth factor (VEGF), proteins that regulate apoptosis (caspase-14) and inflammation (IL-10), and mRNA involved in regulating transcription, cell cycle, and the immune system [9]—can play a protective role in the kidney. Recent studies have shown that miRNAs may also be important mediators of stem cell EVs for organ protection [10]. Alex et al. confirmed for the first time that EVs derived from embryonic stem cells can deliver miRNA to target cells [11]. Since then, in-depth studies have been conducted on the role of stem cell EV miRNAs in target organs. In 2011, Gatti et al. used high concentrations of RNase to pretreat human bone marrow-derived MSCs to inhibit RNA activity and found that the protective effect of MSC EVs on ischemia/reperfusion-induced acute/chronic renal injury in rats was significantly weakened, apoptosis was increased, and proliferation was reduced. These results indicate that miRNAs carried by stem cells derived from EVs play an important role in alleviating renal damage in rats [12]. miRNAs have gradually opened a new direction in the study of protective mechanisms of stem cell EVs in the kidney (Table 1).

**Table 1:** The study of miRNA in stem cell EVs for AKI

Study	Animal	Model	Stem cell source	Route of administration	Method	miRNA	Result
Gatti et al. 2011 [12]	Rat	IRI	Human bone marrow	IV	Pretreatment of MVs with RNase	miRNA	Reduce TECs apoptosis and increase TECs proliferation
Collino et al. 2015 [4]	Rat	Glycerol	Human bone marrow	IV	RNA-Seq, Drosha-Knockdown MSCs	miRNA	Anti-inflammation
Wang et al. 2016 [28]	Mouse	UUO	Human bone marrow	IV	Engineered to overexpress miRNA-let7c	miRNA-let7c	Alleviate kidney fibrosis
Wang et al. 2015 [19]	Mouse	UUO	Mice primary bone marrow	IV	MicroRNA profiling arrays	miRNA	Enhanced inhibition of EMT
Wang et al. 2015 [19]	HK-2	TGF-β1-mediated	Rat bone marrow		miRCURY LNA Array miRNA treatment	miR-133b-3p miR-294	Inhibited TGF-β1-mediated EMT
Yuan et al. 2017 [34]	Mouse	IRI	Mice bone marrow	IV	miR-223 knockdown	miR-223	Downregulation of NLRP3, suppressed apoptosis
Zhu et al. 2019 [36]	Mouse	IRI	Human bone marrow	IV	Overexpression or knockdown of miR-199a-3p	miR-199a-3p	Restore Sema3A expression and blocke the activation of the AKT and ERK pathways, antiapoptosis
Zou et al. 2014	Rat	IRI	Human umbilical cord	IV	CX3CL1 in the TargetScan database, PCR	miR-15a, miR-15b and miR-16	Cell proliferation , Antifibrosis
Gu et al. 2016 [43]	Rat	IRI	Human umbilical cord	IV	miR-30 antagomir treat MSC	miR30	Antiapoptosis, Preserved mitochondrial,morphology
Cantaluppi et al. 2012	Rat	IRI	Endothelial progenitor cells EPC	IV	miRNA array, anti-miR126 and anti-miR-296 antagomiRs	miR-126 and miR-296	Enhancement of cell proliferation,Decrease in tubular cell apoptosis and leukocyte infiltration
Vinas et al. 2016 [51]	Rat	IRI	Human cord blood endothelial colonyforming cells	IV	Truseq Small RNA kit antagomiR to miR-486-5p	miR-486-5p	Target at PTEN/Akt pathway
Pang et al. 2017 [52]	Rat	IRI	Human renal artery-derived vascular progenitor cells	IV	miRNA microarray LNA-miR218	miR-218	Enhance endothelial cell migration

*UUO* unilateral ureteral obstruction, *IRI* ischemia-reperfusion injury, *IV* intravenous, *TEC* tubular epithelial cells, *EMT* epithelial-mesenchymal transition

### miRNAs play important roles in the pathological changes resulting from AKI

Through posttranscriptional gene regulation, miRNAs play important roles in kidney development and physiological conditions as well as pathological conditions. For example, miRNAs are involved in diabetic nephropathy, polycystic kidney disease, hypertensive nephropathy, chronic kidney disease (CKD), and kidney transplantation [13]. In 2016, Dong et al. summarized the miRNAs involved in AKI pathology. Among the two widely used AKI models, kidney ischemia injury and cisplatin-induced toxic injury, at least a dozen miRNAs regulate major pathways involved in inflammation, apoptosis, fibrosis, and the cell cycle, such as phosphatase and tensin homolog (PTEN), heme oxygenase (HO-1), phosphoinositide 3-kinases (PI3K), forkhead box O3 (Foxo3), B cell lymphoma 2 (BCL-2), and hypoxia-inducible factor (HIF)-1, indicating the important role of miRNAs in AKI [14]. In 2018, studies on related topics continued to be published, confirming that cisplatin-induced upregulation of miRNA-709 in an AKI model and inhibition of mitochondrial transcription factor A (TFAM) lead to mitochondrial dysfunction and renal tubular apoptosis. The increased miRNA-709 has also been detected in kidney tissue of patients with AKI with multiple etiologies [15]. These results all indicate the important role of miRNAs in the AKI pathological process.

Almeida et al. performed miRNA microarray detection, gene ontology (GO) analysis, and target gene prediction in kidney tissue with cisplatin-induced AKI and stem cell therapy. They found that the treatment with stem cells involved a substantial miRNA-mRNA regulatory network. For example, miRNA-294, miRNA-141, and miRNA-588 are associated with the Wnt/TGF-β pathways. The authors also speculated that these miRNA changes might be associated with EVs secreted by stem cells [16].

### Protective effect of MSC EV-derived miRNAs on AKI

MSCs are pluripotent stem cells with high self-renewal ability and multidirectional differentiation potential. MSCs have been used to treat myocardial injury and nerve damage diseases in clinical practice. MSCs have been proven to play a therapeutic role in AKI and CKD models. Due to their homing effect, MSCs rarely accumulate in the kidney; therefore, paracrine action may be one of the important mechanisms through which MSCs alleviate kidney diseases. In addition to the release of cytokines, chemokines, and growth factors, paracrine effect is closely associated with the abundant EVs that are secreted by MSCs [17].

In an in vitro model of ATP depletion-induced renal tubular cell injury, Rafael et al. found EV-mediated miRNA delivery from MSCs to renal tubular epithelial cells [18]. Subsequently, they used a glycerol-induced AKI rat model to further clarify the role of EV miRNAs. The double-stranded RNA-specific ribonuclease Drosha is an important enzyme in miRNA synthesis. In the nucleus, RNA molecules generated by the transcription are cleaved by Drosha into precursor miRNAs (pre-miRNAs). Using MSCs with Drosha knockdown, approximately 50 miRNAs in EVs secreted by MSCs were identified as significantly downregulated. Compared with normal MSC and EV treatments, the protective effects of genetically modified MSC-Dsh and EV-Dsh on AKI rats were reduced, as was renal tubular regeneration ability [4]. In the same year, Wang et al. pretreated MSC EVs with erythropoietin (EPO) and found that EPO treatment not only increased the number of EVs secreted from MSC but also significantly improved the inhibitory effect of EVs on fibrosis in a unilateral ureter obstruction (UUO) mouse model and on fibrosis and apoptosis in HK-2 cells after 24 and 48 h induction by TGF-β. A miRNA microarray analysis was used to compare miRNA expression profiles in MSC EVs and EPO-EVs. The miRNA expression profile showed that the expression of 212 miRNAs in EPO-EVs changed significantly (fold-change ≥ 1.5), including miR-299, miR-499, miR-302, and miRNA-200. A total of 70.28% of those miRNA changes were upregulation. Although the authors did not perform knockout validation on upregulated miRNAs, they did indirectly confirm that miRNA alteration in pretreated EVs may underlie enhanced AKI.

### Antifibrosis in UUO

In addition to global miRNA expression profiles, the protective effect of MSC EV-derived miRNAs on AKI has also been studied for specific miRNAs. In in vitro experiments, TGF-β was used to induce epithelial-mesenchymal transition (EMT) in HK-2 cells. Bone marrow-derived MSC EVs from young and aged rats were given to HK-2. The results showed that the inhibitory effect of MSC EVs from aged rats on EMT was significantly reduced. By comparison, they found that the expression of miR-344a, miR-133b-3p, miR-294, miR-423-3p, and miR-872-3p in MSC EVs of aged rats was significantly lower than that in young rats; the serum miRNA levels showed the same patterns. Direct intervention of TGF-β-induced HK-2 cells with the 4 miRNAs showed that miR-133b-3p and miR-294 significantly inhibited EMT in HK-2 cells [19]. miR-133b also played a protective role in other organs with effects such as inhibition of myocardial remodeling [20], improved cerebral ischemia [21], and reduced spinal cord injury [22]. However, some studies have found that knockdown of miR-133b and miR-199b alleviates diabetic nephropathy, renal fibrosis, and TGF-β1-induced EMT through sirtuin 1 (SIRT1) [23]. Therefore, the function of miR-133 in kidney fibrosis needs to be further investigated.

In 2000, a study in *Nature* first reported the regulatory effect of let-7 miRNAs on nematode development [24]. Let-7 is highly conserved among multiple species [25] and may play a protective role in kidney fibrosis. JASN published an article in 2013 reporting that lipoxin-induced let-7c can reduce renal fibrosis through inhibition of TGFβR1, demonstrating for the first time the important role of let-7 in kidney fibrosis [26]. Wang et al. conducted a series of studies on the relationship between let-7 and kidney injury. In diabetic and nondiabetic renal fibrosis models, let-7b expression decreases as TGFβR1 increases. Let-7b can directly inhibit the expression of TGF-β1 receptor 1 (TGF-7b), reduce extracellular matrix proteins, decrease the activity of TGF-β1, and reduce the profibrotic effect of TGF-β1 on normal rat kidney epithelial cells NRK52E through binding targeted genes [27]. Subsequently, they used gene editing technology to overexpress let-7c in human bone marrow which resulted in a significant increase in let-7c in MSC EVs. Fluorescence microscopy was used to observe the transport of EVs to NRK52E cells, revealing increased expression of let-7c in NRK52E cells. After GW4869 treatment to inhibit EV production, the aforementioned changes and inhibitory effects on fibrosis all weakened. MSCs overexpressing let-7c also exert a better therapeutic effect on UUO [28].

### Anti-inflammatory and anti-apoptosis effects in ischemia-reperfusion injury (IRI)

Inflammation plays an important role in the pathophysiology of AKI. Various specific inflammatory cells and pro-inflammatory cytokines/chemokines increase in the kidney during early AKI caused by ischemia reperfusion [29]. The chemokine C-X3-C motif chemokine ligand 1 (CX3CL1), a macrophage chemokine, is mainly expressed in endothelial cells. Inhibition of CX3CR1 can reduce macrophages in injured kidneys and has therapeutic effects on AKI [30]. Therefore, CX3CL1 is an important anti-inflammatory target for the treatment of renal IRI. Wharton’s jelly is another important source of MSCs other than the bone marrow. When Zou administered human Wharton’s jelly MSC (hWJMSC) EVs to IRI rats, apoptosis was alleviated, proliferation was enhanced, and the inflammatory response was alleviated at 48 h in the kidney. Additionally, hWJMSC EVs inhibited CX3CL1 expression and reduced the number of CD68+ macrophages in the kidney. By searching for miRNAs predicted to target CX3CL1 in the TargetScan database, multiple miRNAs (miR-15a, miR-15b, miR-16, miR-195, miR-424, and miR-497) that might be involved in the regulation were discovered. Finally, RT-PCR analysis showed that hWJMSC EVs contained higher levels of miR-15a, miR-15b, and miR-16. However, the author did not further validate these 3 miRNAs. NLR family-pyrin domain containing 3 (NLRP3) inflammasomes promote inflammatory cytokine production during kidney injury and are highly expressed after renal tubular injury [31]. miRNA-233 can regulate NLRP3 expression [32]. The protective effect of MSC EVs on the heart is also associated with miRNA-233 [33]. Hypoxia preconditioning significantly increased miRNA-233 levels in MSCs and inhibited hypoxia/reoxygenation (H/R)-induced renal tubular cell apoptosis. A dual luciferase assay showed that miR-223 directly inhibited NLRP3 expression and activated Notch1. After knockdown of miRNA-233 in MSCs, H/R-induced renal tubular apoptosis was significantly increased. In vivo experiments also showed that the protective effect of MSCs on renal ischemia injury significantly decreased after miRNA-233 knockdown [34]. Although this study only used MSC therapy without EVs, miRNA-233 in EVs may be one of the mechanisms by which MSCs exert their therapeutic effect. The miRNAs in exosomes secreted from human bone marrow MSC EVs consisted of many miRNAs and miR-199a-3p was the most abundant. It has been found that exosomes loaded with a high dose of miR-199a could prevent cardiomyocyte apoptosis [35]. MSC exosome treatment could also increase the expression level of miR-199a-3p in renal cells and prevent IRI injury of the kidney. According to the prediction of target gene, Sema3A, a member of class 3 semaphorins, mediated the effect of miR-199a-3p. The activation of Sema3A could suppress the protein kinase B (AKT) and extracellular signal-regulated kinase (ERK) pathways, which are related to cell survival [36].

In addition to inflammatory responses, studies have shown that mitochondrial dynamics also contribute to pathological changes after renal IRI [37]. Under IRI-induced stress, mitochondria dynamics shift to the dividing state, eventually leading to mitochondrial disruption and apoptosis [38]. Dynamin-related protein 1 (Drp1) is a key factor affecting mitochondrial division. Inhibition of Drp1 can block mitochondrial rupture and protect renal ischemia or cisplatin-induced acute injury [39]. miRNA-30 regulates mitochondrial division in cardiomyocytes through Drp1 [40]. Additionally, miR-30 also imparts renal protection through inhibition of apoptosis [41] and fibrosis [42]. However, the relationship with mitochondria dynamics has not been studied extensively in the kidney. Gu et al. observed injury and mitochondrial morphology in IRI rats treated with hWJMSC EVs and found that the expression of miRNA-30 in the kidneys of the treatment group rats increased, DRP1 expression decreased, mitochondria returned to a normal state, and apoptosis was reduced. After antagomiR was used to reduce miRNA-30 expression in MSC EVs, the therapeutic effect of EVs was significantly reduced [43]. These results suggest that miRNA-30 in EVs protect mitochondrial morphology and apoptosis in the kidney by regulating DRP1.

### Therapeutic effect of miRNAs on AKI in progenitor cell EVs

In 1997, Asahara isolated circulating endothelial progenitor cells (EPCs). This CD34+ cell type contributes to angiogenesis [44]. EPCs circulate in the peripheral blood and can home in on injured tissues and organs, release angiogenic factors and EVs, stimulate local endothelium, and differentiate into mature endothelial cells that incorporate into injured tissue to replace or support the existing endothelium, form new blood vessels, and promote vascular repair and regeneration [45]. Previous studies also confirmed that EPCs have therapeutic effects on AKI [46].

Dicer is an endonuclease that belongs to the RNase III family and specifically recognizes double-stranded RNA. Dicer digests pre-miRNA to form mature miRNA. Cantalupi et al. found that exogenously administered EPC EVs colonize glomeruli and renal tubules after renal IRI. Dicer knockout eliminates miRNAs in EPCs, which reduces the protective effect of EVs on renal IRI. Finally, it was found that miR126 and miR-296 might mediate EPC EV actions through promoting cell proliferation and reducing apoptosis and inflammatory cell infiltration [47]. Previous studies demonstrated that miRNA-126 regulates the PI3K pathway, regulates cell regeneration, and plays a protective role in the kidney [14]. Studies have shown that miR-296 is highly expressed in aged F344 rats, and pathway analysis revealed that it may be associated with inflammation in kidneys of elderly individuals [48]. However, this study was not verified. Additionally, the miRNA expression profile analysis of F344 rats was not representative. AKI is a type of acute injury, while kidney damage in elderly individuals may result from chronic processes. miR-296 may play different roles at different stages. In the future, miR-296 should be studied further by establishing an acute and chronic kidney model for the elderly.

Endothelial colony-forming cells (ECPCs) are one type of EPC [49]. A series of studies by Burger and Viñas on ECPCs also confirmed that in hypoxic/reoxygenation (H/R) human umbilical vein endothelial cells and an IRI rat model, ECPC EVs alleviate renal cell oxidative stress-induced injury and cell apoptosis [50]. Subsequently, microRNA-486-5p in ECPC EVs were found to inhibit epithelial cell apoptosis by regulating the PTEN/Akt pathway [51].

miRNAs in EVs can play a protective role in the AKI process, while inhibiting miRNAs in EVs may reduce renal tissue damage. Pang et al. isolated CD34+/CD105− renal artery-derived vascular proliferative cells (RAPCs) from the renal artery of patients during radical nephrectomy. These cells showed EPC-like characteristics as they integrated into glomerular capillaries to increase the capillary density in the kidneys of ischemic rats. After inhibiting the expression of miRNA-218 in RAPCs, the RAPC EVs had enhanced promotion of endothelial cell migration. This may be because the inhibition increased roundabout protein (Robo-1) mRNA in EVs, and Robo-1 further migrated to the kidney during treatment to promote endothelial migration [52]. Robo-1 can contribute to the regulation of angiogenesis and endothelial cell migration.

Other stem cell EVs, such as stem cells isolated from urine, liver-derived stem cells, and glomerular stem cells, also play protective roles in acute and chronic kidney injury [53]. However, the role of miRNAs in EVs secreted by these stem cells has not yet been studied in depth.

## Conclusions

In summary, EVs secreted by stem cells play important roles in the injury and repair of AKI through miRNAs, mainly through anti-fibrotic, anti-inflammatory, and anti-apoptotic functions in UUO and ischemic injury. They can also promote tissue repair and regeneration. In the future, miRNAs may not only be used biomarkers for AKI injury but also as markers of stem cell treatment efficacy. However, to date, there are few studies on which miRNAs and associated target genes mediate the therapeutic effect of stem cell EVs on AKI. In addition to the studies covered in this review, other miRNAs that play a role remain to be determined. The main sources of EVs are currently concentrated in MSCs and EPCs, and the protective effect of other stem cell-derived miRNAs on AKI needs to be further clarified. miRNAs may play different roles during different stages of the physiology and pathology of the kidney. In summary, miRNA studies on EVs in stem cells provide important reference for clinical practice and provide new ideas for finding more effective and direct treatment strategies and therapeutic targets for acute and chronic kidney diseases.

## Data Availability

Not applicable.

## Abbreviations

AKI: Acute kidney injury
BCL-2: B-cell lymphoma 2
CKD: Chronic kidney disease
CX3CL1: C-X3-C motif chemokine ligand 1
Drp1: Dynamin-related protein 1
ECPCs: Endothelial colony-forming cells
EPO: Erythropoietin
EVs: Extracellular vesicles
Foxo3: Forkhead box O3
H/R: Hypoxia/reoxygenation
HGF: Hepatocyte growth factor
HIF: Hypoxia-inducible factor
HO-1: Heme oxygenase
hWJMSC: Human Wharton’s jelly MSC
miRNA: MicroRNA
MSCs: Mesenchymal stem cells
NLRP3: NLR family-pyrin domain containing 3
PI3K: Phosphoinositide 3-kinases
PTEN: Phosphatase and tensin homolog
RAPCs: Renal artery-derived vascular proliferative cells
Robo-1: Roundabout protein
TFAM: Mitochondrial transcription factor A
TGFβR1: Transforming growth factor-β receptor type 1
UUO: Unilateral ureter obstruction
VEGF: Vascular endothelial growth factor
